# Imaging modalities used in diagnosis and follow-up of patients with Takayasu’s arteritis

**DOI:** 10.3906/sag-2005-70

**Published:** 2021-02-26

**Authors:** Ayşe Bahar KELEŞOĞLU DİNÇER, Levent KILIÇ, Abdulsamet ERDEN, Umut KALYONCU, Tuncay HAZIROLAN, Sedat KİRAZ, Ömer KARADAĞ

**Affiliations:** 1 Department of Internal Medical Sciences, Faculty of Medicine, Hacettepe University, Ankara Turkey; 2 Department of Rheumatology, Faculty of Medicine, Hacettepe University, Ankara Turkey; 3 Department of Radiology, Faculty of Medicine, Hacettepe University, Ankara Turkey

**Keywords:** Takayasu’s arteritis, computer tomography angiography, magnetic resonance angiography, doppler ultrasonography, conventional angiography, positron-emission tomography

## Abstract

**Background/aim:**

Takayasu’s arteritis (TA) is a rare, large-vessel vasculitis of unknown etiology, affecting aortic arch, and its main branches. Noninvasive imaging methods are frequently used in diagnosis and follow-up in Takayasu’s arteritis. Studies investigating optimal timing of follow up imaging are rare. This study is aimed to investigate the radiologic changes in vascular involvements of Takayasu’s arteritis patients one year after diagnosis.

**Materials and methods:**

Database of our Vasculitis Center was analyzed retrospectively and 97 patients were included into the study. Demographic, clinical, radiological, and therapeutic findings of patients were recorded. Patients with follow-up imaging after approximately one year of diagnosis were recruited into further analysis. Radiological changes and the effect of different immunosuppressive agents on vascular involvements were investigated.

**Results:**

Mean age and disease duration of patients were 43.0 and 9.0 years. The most commonly used imaging methods/modalities for the diagnosis of TA were computer tomography-angiography (CT-Ang) (58.8%), magnetic resonance-angiography (MR-Ang) (29.9%), and doppler ultrasonography (11.3%). Subclavian and common carotid arteries were the most frequently involved vessels. Fifty-three patients underwent follow-up imaging after one year of diagnosis and, in 64% of patients, same imaging method had been used. MR-Ang (62.3%) and CT-Ang (35.9%) were the most preferred follow-up imaging studies. Sixty-eight percent of patients had stable vascular involvement, 28% had progression, and 4% had regression. No difference was found in radiological changes regarding patients with usage of different immunosuppressive agents (P = 0.634). There was no association between the change in serum acute phase reactants and radiological disease activity.

**Conclusion:**

The most commonly used imaging modality for the diagnosis of TA was CT-Ang, whereas MR-Ang was the most preferred for follow-up. Almost 30% of TA patients in our Vasculitis Center had progression at around one year concordant with previous literature. A follow-up imaging at around one year of treatment seems feasible in management of TA.

## 1. Introduction 

Takayasu’s arteritis is a rare, large-vessel vasculitis of unknown etiology, characterized by granulomatous inflammation of the vessel wall [1]. It frequently involves the aortic arch and its main branches, the ascending aorta, the thoracic descending aorta, and the abdominal aorta [1]. 

Noninvasive imaging methods such as magnetic resonance-angiography, computer tomography-angiography, Doppler ultrasonography and 18F-fluorodeoxyglucose positron emission tomography/computed tomography (PET/CT) have replaced conventional angiography for diagnosis and monitoring of disease activity in TA [1]. These noninvasive imaging methods are superior to conventional angiography because they can detect both luminal changes and early inflammatory signs of the vessel wall (thickening of the vessel wall and mural inflammation) and late complications (stenosis and aneurysm) [2]. However, there is an uncertainty about which imaging method will be chosen for diagnosis and follow-up of patients with large vessel vasculitis.

The aim of this study is to evaluate the used imaging modalities for diagnosis and follow-up of TA patients.

## 2. Materials and methods

### 2.1. Study population

In the prospective database of the Hacettepe University Vasculitis Research Center (HUVAC), 97 TA patients meeting the 1990 modified American College of Rheumatology (ACR) criteria [3] were registered by the end of July 2017. In the second step, 53 TA patients who had a follow-up imaging study approximately one year after the diagnosis were enrolled in the subsequent analysis.

### 2.2. Demographic and clinical features 

Data about demographics, comorbidities, disease duration, distribution of vascular involvement, laboratory, previous and current medications were obtained from hospital records. Clinical disease activity was characterized by new vascular/ischemic signs or general symptoms occurrence, or inflammatory laboratory markers elevation. Erythrocyte sedimentation rate (ESR) (0-20 mm / h) and C-reactive protein (CRP) levels (<1 mg/dL) were used as markers of disease activity. 

### 2.3. Imaging modalities

Type of imaging methods (Doppler US, MR-Ang, CT-Ang, conventional angiography and PET/CT) used for diagnosis and to monitor the disease activity approximately one year ( ± 3 months) after the diagnosis were obtained from the hospital records. In addition, we have recorded in detail the distribution of involved vascular areas in radiological imaging (one reader exists). The angiographic findings were divided into five groups as described by the International Conference on Takayasu Arteritis in Tokyo in 1994: type 1 involves the aortic arch branches; type 2a involves the ascending aorta, arch and its branches; type 2b involves the ascending aorta, arch with its branches, and the descending aorta; type 3 involves the thoracic descending aorta, abdominal aorta, and/or renal arteries; type 4 involves only the abdominal aorta and/or renal arteries; type 5 is the combined features of type 2b and 4 [4]. 

### 2.4. Follow up of data regarding acute phase reactants and radiologic evaluation

The change in acute phase reactants was described according to their cut-off levels (i.e; ESR<20 mm/h, CRP <1mg/dL). If the initial level was above but the follow-up level was below the cut-off point, it was given as a decrease in acute phase reactant level. If the initial level was lower and the follow-up level was higher than the cut-off, it was given as an increase. If both initial and follow-up levels were higher or lower than the cut-off levels, then it was given as no change in acute phase reactants.

Radiological disease activity was classified as stable disease (absence of any worsening or improvement in stenotic or dilatated vasculitic lesions), progression (development of a new lesion: vessel wall thickening/irregularity, stenosis, occlusion, aneurysm, and dilatation or worsening of the preexisting stenosis or dilatation) and regression (improvement of preexisting vasculitic lesions) according to follow-up imaging studies.

### 2.5. Ethical considerations

The study protocol was approved by Institutional Ethics Committee (20.02.2014/GO14/84-16).

### 2.6. Statistical analysis

Statistical analyses were performed using the SPSS software version 22 (IBM Corp., Armonk, NY, USA). The variables were investigated using visual (histograms, probability plots) and analytical methods (Kolmogorov-Simirnov/Shapiro-Wilk’s test) to determine whether or not they are normally distributed. Descriptive analyses were presented using means and standard deviations for normally distributed variables, medians, and interquartile ranges for the nonnormally distributed variables, frequencies for the categorical variables. Chi-square test was used to analyze categorical variables. In the comparisons between groups, the paired-samples t-test was used for normally distributed variables and the Wilcoxon for nonnormally distributed variables. A P-value of less than 0.05 was considered to show a statistically significant result.

## 3. Results

### 3.1. Demographic, clinical, and treatment data

We recruited ninety-seven TA patients (92.8% female). The demographic and clinical characteristics of TA patients were summarized in Table 1. None of the patients received a biological agent for first-line therapy. Twenty-four patients (24.7%) switched either to another conventional immunosuppressive agent (n = 11) or to a biological agent (n = 13). The most commonly used biological agents were tocilizumab (n = 5), infliximab (n = 4), adalimumab (n = 2), and etanercept (n = 2), respectively.

**Table 1:** Demographic, laboratory, and treatment features of patients with Takayasu’s arteritis (n = 97).

Female, (%) (n)	92.8% (90)
Age, years ϯ	43.0 ± 14.4
Age at diagnosis,years ϯ	34.7 ± 12.1
Disease duration, years ϯ	9.1 ± 5.2
Diagnosis delay time,months (median,IQR)	19.0 (36)
ESR (mm/h) ϯ	37.3 ± 28.6
CRP (mg/L) ϯ	31.0 ± 48.0
Hemoglobin (g/dL) ϯ	12.2 ± 1.6
Mean corpuscular volume ϯ (fL)	81.0 ± 7.5
White blood cell count ϯ (103/ mm3)	9.347 ± 3.800
Thrombocyte count ϯ (µL)	357.019 ± 110.940
Treatment features	
Corticosteroids, n (%)	97 (100%)
Methotrexate, n (%)	38 (39.2%)
Cyclophosphamide, n (%)	36 (37.1%)
Azatioprine, n (%)	21 (21.6)
Micophenolate mofetile, n (%)	2 (2.1)
Biological drugs, n (%)	13 (13.4%)

ϯ Values are mean ± standard deviation.

### 3.2. Imaging modalities and angiographic characteristics

The imaging modalities performed for diagnosis of TA were CT/CT angiography in 57 (58.8%) patients, MR angiography in 29 (29.9%) patients, and Doppler US examination in 11(11.3%) patients. (Table 2) In the initial diagnosis, the most frequently affected vessels were left subclavian artery (67.9%), left common carotid artery (52.8%), right subclavian artery (47.2%), and aortic arch (47.2%), respectively. (Table 3) According to the 1994 angiographic classification system, the most common type of angiographic involvement at diagnosis was type V (39.2%), followed by type I (26.8%), type IIb (16.5%), type IIa (11.3%) and type IV (2.1%). Pulmonary arterial involvement was similar in both right and left pulmonary arteries (11.3%). Renal artery stenosis was detected in 20.8% of patients in the left renal artery and in 9.4% of patients in the right renal artery (Table 3). 

**Table 2:** Angiographical findings of TA patients at diagnosis.

Imaging modality	n(%)
CT- Ang	57 (58.8)
MR-Ang	29 (29.9)
Doppler US	11 (11.3)
PET-CT	0 (0)
Conventional angiography	0(0)
Lesion type	n(%)
Type 1	26 (26.8)
Type 2a	11 (11.3)
Type 2b	16 (16.5)
Type 3	4 ( 4.1)
Type 4	2 (2.1)
Type 5	38 (39.2)

**Table 3:** The frequencies of vascular involvements of TA patients.

Vascular involvement	(%)
Ascending aorta	37.7
Aortic arch	47.2
Descending Thoracic aorta	43.4
Abdominal aorta	45.3
Left subclavian	67.9
Left common carotid artery	52.8
Truncus brachiocephalicus	22.6
Right subclavian artery	47.2
Right common carotid artery	37.7
Right axillary artery	17.0
Left axillary artery	15.1
Right iliac artery	9.4
Left iliac artery	9.4
Right femoral artery	3.8
Left femoral artery	3.8
Right renal artery	9.4
Left renal artery	20.8
Right pulmonary artery	11.3
Left pulmonary artery	11.3

### 3.3 Follow up of data regarding acute phase reactants and radiologic evaluation

A follow-up imaging study was scheduled for 53 (n = 50 females) patients approximately one year after the diagnosis. Imaging techniques used to assess disease activity during follow-up were MR-Ang (62.3%), CT-Ang (35.8%), and Doppler US (1.9%) (Table 4). In 34 of patients (64%), initial diagnostic and follow-up imaging modalities were the same. Follow-up studies showed radiologically stable disease in 32 patients (60.4%), progression in 15 patients (28.3%), and regression in six patients (11.3%). (Table 5) In six of 15 patients (40%) with radiological progression, treatment was modified. 

**Table 4:** Imaging methods used at diagnosis and during follow-up of TA patients (n = 53).

Imaging method	Diagnosis n (%)	Follow-up	n (%)
Conventional-ang	0 (0)		0 (0)
Doppler US	6 (11.3)	Doppler USMR-ang	1 (16.7)5 (83.8)
CT angiography	31 (58.5)	CT-angMR-ang	18 (58.1)13 (41.9)
MR angiography	16 (30.2)	CT- angMR-ang	1 (6.2)15 (93.8)

**Table 5:** Association of change in acute phase reactants and initial treatment features of TA patients with radiological disease activity status at follow-up (n = 53).

	Radiologically Stable,n(%)	Radiologically Progression,n(%)	RadiologicallyRegression,n(%)	P value
Change in ESR ϯ				0.177
Increase	2 (6.3)	1 (6.7)	1 (16.7)	
Decrease	13 (40.6)	2 (13.3)	3 (50.0)	
No change	17 (53.1)	12 (80.0)	2 (33.3)	
Change in CRP ϯ				0.375
Increase	5 (15.6)	3 (20.0)	0 (0.0)	
Decrease	9 (28.1)	6 (40.0)	4 (66.7)	
No change	18 (56.3)	6 (40.0)	2 (33.3)	
Initial treatment ϯ				0.614
CYC	11 (34.3)	6 (40.0)	2 (33.3)	
MTX	12 (37.5)	6 (40.0)	4 (66.7)	
AZA	8 (25.0)	3 (20.0)	0 (0.0)	
MMF	1 (3.1)	0 (0.0)	0 (0.0)	

AZA: Azatiopyrin; CYC: Cyclophosphamide; MMF: Mycophenolat mofetil; MTX: Methrotrexate. ϯChi-square test was used.

When the association between the radiological disease activity and the change in acute phase reactants at the time of follow-up was evaluated, no association was found between the groups. (Table 5) At the time of follow-up imaging, 71.2% of patients had normal ESR and CRP levels even though these patients had elevated acute phase reactants at diagnosis. In patients with radiologically stable disease, five patients (15.6%) had elevated CRP levels at the time of follow-up imaging although their initial levels were within normal levels. Nine patients with high baseline CRP were found to regress to normal limits during follow-up imaging. In eight of 15 patients with radiologic progression, the ESR and/or CRP levels were found within normal levels. We did not find significant association between initial treatments of patients and the radiological disease activity status at follow-up. (Table 5) When the changes in acute phase reactants at diagnosis and approximately one year follow-up were evaluated, we found a statistically significant decrease (P < 0.001). The changes in acute phase reactants and hematological parameters were summarized in Table 6.

**Table 6:** Laboratory features of TA patients at diagnosis and during control imaging (n = 53).

	Diagnosis	Follow-up	P
ESR (mm/h), median (IQR)	39.5 (12.3-59.8)	15.5 (9.3-24)	<0.001
CRP (mg/L) , median (IQR)	1.61 (0.74-4.7)	0.64 (0.37-1.32)	<0.001
Hemoglobin (g/dL), mean (± SS)	12.2 ± 1.6	12.7 ± 1.7	0.066
MCV (fL) , mean (± SS)	81 ± 7.5	84.2 ± 7.5	<0.001
WBC (103/mm3) , median (IQR)	9100(7050-11100)	9000(6750-12200)	0.431
Thrombocyte count(µL), mean (± SS)	357019 ± 110940	329340 ± 78549	0.053

Hb: Hemoglobin; MCV: Mean corpuscular volume; SD: Standard deviation; WBC: White blood cell. ϯCorrelation between repeated measures were analyzed by Wilcoxon and paired-samples t test.

## 4. Discussion

In recent years, imaging methods such as MR-Ang, CT-Ang, and PET/CT have become important in large-vessel vasculitis [2]. These methods offer a more sensitive and rapid noninvasive assessment of aorta, cranial, and extracranial arteries compared to conventional angiography [2]. Although these new imaging methods have many advantages, there is an uncertainty about which imaging method will be chosen for diagnosis and follow-up of patients. In this study, CT-angiography was the most commonly used imaging methods for diagnosis, whereas MR-Angiography was the most commonly used imaging method for follow-up. The European League Against Rheumatology (EULAR) 2018 guidelines on the imaging of large vessel vasculitis suggested MR-Ang as the first-line imaging modality for the diagnosis of TA due to the lack of radiation exposure and it is reported as an expert’s opinion that CT, PET, and/or US can also be used as alternative imaging modalities [2]. 

In 1994, Takayasu’s arteritis was classified into five groups according to angiographic findings [4]. In our study, types V and I had the highest frequency followed by types IIa, IIb, III and IV, respectively. A cohort by Ohigashi et al. in Japan, in accordance with our study, reported the highest frequency in type 5, followed by type 1, with the lowest frequency in type 4 [5]. The study conducted by Freitas et al. reported the highest frequency in types V and I followed by types IV, III, IIa, and IIb, respectively [6]. In TA, the subclavian artery is the most frequently involved vessel and the left subclavian artery is more frequently involved than the right [7]. The aorta, the main carotid artery, the renal artery and the other primary branches of the aorta have lower rates of involvement, respectively [8]. In our patients, the left subclavian artery had the highest frequency (67.9%) of involvement, followed by left main carotid artery, right subclavian artery, aortic arch, abdominal aorta, and descending aorta, respectively. Similarly, a cohort of 248 patients from Turkey showed that the left subclavian artery had the highest frequency of involvement (76.0%), followed by carotid arteries (52.0%) [9]. However, Freitas et al. from Brasil reported the highest frequency of involvement in the abdominal aorta, followed by the left subclavian artery and the left main carotid artery, respectively [6]. The difference in the involvement of arterial lesions in different countries can be attributed to the role of ethnic and genetic factors in the pathogenesis of TA [5]. Pulmonary artery involvement in TA is seen up to 50.0% of patients [10]. In the study of Sharma et al. with 44 TA patients, pulmonary artery involvement was seen in 14.3% of patients [11]. In our study, though, we found pulmonary artery involvement in 11.3% of the patients. Renal artery involvement is reported to be seen in 8%-38% of TA patients [12]. Renal artery involvement was recorded in 20.8% of our patients in the left renal artery, in 9.4% in the right renal artery, and, in 28.6% of them, bilateral involvement was seen. 

There is also uncertainty about the timing of follow-up imaging studies to assess disease activity and the degree of damage. Chatterjee et al. suggested that follow-up imaging studies should be performed in the presence of suspected relapse or at least annually in asymptomatic disease [13]. The Cleveland Clinic Center for Vasculitis Care and Research has been performing imaging for follow-up of TA patients every 6 to 12 months in routine practice since 1992 [14]. Tombetti and Mason stated that response to treatment of TA is typically monitored by MR-Ang or CT-Ang every 6-12 months for the first two years, and once clinical remission is sustained, annual monitoring by MR-Ang is recommended [15]. On the other hand, 2018 EULAR guidelines stated that follow-up imaging studies should be repeated in the presence of a suspected exacerbation, but that imaging studies are not required in cases with clinical or laboratory remission [2]. The frequency of imaging assessments was recommended to be decided on individual basis. Again, according to this guideline, the type of imaging method for monitoring disease activity in TA and Giant cell arteritis (GCA) should be decided according to expertise and vessels affected [2]. In our study, 53 patients (54.6%) were found to have follow-up imaging studies approximately one year after diagnosis, which was consistent with the literature. MR-Ang (62.3%) and CT-Ang (35.9%) were the most preferred follow-up imaging studies, respectively. There are no standardized and reliable methods for monitoring disease activity and evaluating the therapeutic response in patients with TA. Acute phase proteins are not always correlated with inflammatory activity in the vessel wall [16]. Despite normal ESR and serum CRP levels, active disease in the vessel wall can be detected by imaging methods [14,17,18]. In the second step of our study, we compared radiological disease activity and its correlation with acute phase reactants according to a comparative evaluation of baseline and control imaging studies. In our study, the same imaging modality was used in 64% of patients. Even though, 36% of patients had undergone different imaging modalities for evaluation of vascular involvement, as both MR-Ang and CT-Ang directly reflect hemodynamic disturbance and the risk of end-organ ischemia, comparable data can be obtained from these two imaging modalities [19]. In the study of Tombetti et al., similar to our study, baseline scans of 131 large vessel vasculitis patients (96 TA, 35 LV-GCA) were 114 MR-ang and 17 CT-Ang and 67 patients underwent additional scan of MR-ang at median interval of 18 months [19]. Same approach was also used by Nakagomi et al. in order to develop combined arteritis damage score where they analyzed the angiographic data from CT and MR-Ang of 41 TA and 55 GCA patients retrospectively [20]. In our study, follow-up imaging evaluations showed stable disease in 60.4%, progression in 28.3%, and regression in 11.3% of patients. There was no significant association between radiological findings and change in serum ESR and CRP levels (P = 0.177 and P = 0.375, respectively). An increase in serum ESR levels were found in 6.3% of patients with radiological stable disease (n = 2) and in 6.7% of patients with radiological progressive disease (n = 1). Also, an increase in serum CRP levels were found in 15.6% of patients with radiological stable disease and in 20.0% of patients with radiological progressive disease. In the study of Tombetti, longitudinal MR-Ang of patients of a median of 18 months revealed 40% stable vasculitic lesions, 37% progression, and 23% improvement [19]. Maksimowicz-McKinnon showed normal levels of acute phase reactants in 23% of TA patients with active disease [14]. While ESR and CRP levels were normal in 46% of patients with newly developed vascular involvement in MR angiography, there was an increase in the levels of acute phase reactants in the remaining patients despite no change in MR angiographic examination findings [13].

Evaluating the disease activity of TA is still challenging. [21] There is no standardized assessment of vascular imaging modalities such as CT-Ang, MR-Ang, or PET/CT, and also there is no accepted gold standard for describing radiological assessment [15,18]. Previous studies showed progression of vascular lesions and presence of histologically active disease in half of patients despite clinical and laboratory remission. On the other hand, TA is generally slowly progressive disease and progression rate of radiological findings are variable [22].

The major limitations of our study are as follows: due to retrospective design of the study, disease activity indexes such as Indian Takayasu Arteritis Activity Score (ITAS2010) has not been calculated [23]. Also the data was based on one reader report for each imaging; there is no intra/inter-observer assessment for the radiologic evaluation. 

Additionally, we had six patients diagnosed just with Doppler US and no other modalities. Regarding these six patients, it was difficult to evaluate thoracic, ascendant, and descending aorta, pulmonary arteries, as well. Therefore, convenience of initial classification with doppler US could be limited. 

In conclusion, there is no standard imaging method (MR-Ang, CT-Ang, Doppler US, PET/CT) for assessing disease activity and response to treatment. Although, comparable angiographic data can be obtained from MR-Ang and CT-Ang, their features are different and these imaging modalities cannot be accepted as equivalent as far as inter-modality reproducibility is studied. Therefore, it is usually recommended to use the same imaging technique for both diagnosis and monitoring disease activity. Even though, the data about the frequency of screening disease activity is limited, it seems reasonable to screen patients in whom a flare is suspected or annually in asymptomatic patients. 

## Informed Consent

The study protocol was approved by Hacettepe University Ethics Committee (20.02.2014/GO14/84-16).
